# Silicon Speciation and Its Relationship with Carbon and Nitrogen in the Sediments of a Macrophytic Eutrophic Lake

**DOI:** 10.3390/toxics13040266

**Published:** 2025-03-31

**Authors:** Yong Liu, Guoli Xu, Guocheng Wang, Haiquan Yang, Jv Liu, Hai Guo, Jiaxi Wu, Lujia Jiang, Jingfu Wang

**Affiliations:** 1 College of Biological and Environmental Engineering, Guiyang University, Guiyang 550005, China; 2 State Key Laboratory of Environmental Geochemistry, Institute of Geochemistry, Chinese Academy of Sciences, Guiyang 550081, China; 3 Guizhou Province Field Scientific Observation and Research Station of Hongfeng Reservoir Ecosystem, Guiyang 551499, China

**Keywords:** biogeochemical cycle, macrophytic eutrophic lake, organic matter, silicon species, sediments

## Abstract

Silicon (Si) is one of the biogenic elements in lake aquatic ecosystems. Sediments are both sinks and sources of Si, but little is known about its influence on the biogeochemical cycle of Si in lakes and its relationship to other biogenic factors such as carbon and nitrogen. Examining Caohai Lake, a typical macrophytic lake in China, this study systematically examined the different Si forms and biogenic silica (BSi) distribution characteristics and their coupling relationships with total organic carbon (TOC) and total nitrogen (TN) in surface sediments. Iron–manganese-oxide-bonded silicon (IMOF-Si) and organic sulfide-bonded silicon (OSF-Si) jointly accounted for 95.9% of Valid-Si in the sediments, indicating that the fixation of Si by organic matter and iron–manganese oxides was the main mechanism underlying the formation of the different forms of Valid-Si in sediments. The release and recycling of Si in sediments may be mainly driven by mineralized degradation of organic matter and anoxic reduction conditions at the sediment–water interface. The content of biogenic Si (BSi) in the sediments was relatively higher in the southern and eastern areas, which could be explained by the intensification of eutrophication and the increased abundance of diatomaceous siliceous organisms in these areas seen in recent years. The TOC and TN contents in the sediments were generally high, and the sources of organic matter in the sediments included both the residues of endophytes (main contributors) and the input of terrigenous organic matter. TOC and TN both had highly significant correlations with OSF-Si and Valid-Si, which demonstrated that Valid-Si had excellent coupling relationships with C and N in the sediments. The good correlation between BSi, TOC and TN (*p* < 0.01), as well as the high C/Si, N/Si mole ratio of TOC and TN to BSi, respectivelny, indicating that the dissolution and release rate of BSi may be much higher than the degradation rate of organic matter from the sediments, especially in the areas with a higher abundance of siliceous organisms.

## 1. Introduction

Silicon (Si) is a major element in nature, existing mostly as silicate or silica minerals in rocks and their weathered products such as quartz sand and dust [1,2]. It is also one of the important elements for living substances [3]. In agriculture, Si fertilizers can increase crop yield, contribute to pest control, and improve soil micro-environments [4,5]. In water, Si, like carbon and nitrogen, is an essential biogenic element for lakes, reservoirs, and other aquatic ecosystems [6,7,8]. As it is often much higher in concentration than carbon and nitrogen, Si experiences very complex biogeochemical cycles [9,10]. Si in lakes initially mainly results from the weathering of terrigenous particulate Si or the runoff input of dissolved Si [9,11]. The majority of insoluble Si exists in the form of suspended fine or coarse particles that can be gradually or rapidly deposited, while a small portion exists in the form of dissolved H_4_SiO_4_ [12]. It can be adsorbed, fixed, and co-deposited by carbonates, metal oxides, and organic matter, forming different forms in sediments [13,14,15,16]. Dissolved Si in the water can be directly absorbed and utilized by aquatic organisms, such as diatoms, sponges, radiolaria, silicoflagellates, dinoflagellates, and cyanobacteria; when these siliceous organisms die, they deposit it to form biogenic silica (BSi) [15,17,18]. Thus, sediments constitute the main sink of Si [19].

In general, Si in sediments can be either valid (Valid-Si) or non-valid silicon (Non-valid-Si). The vast majority of Non-valid-Si exists in the form of indissolvable silicate or silica minerals, which barely influence biogeochemical cycles. Valid-Si, which accounts for a relatively small portion (usually less than 10%), often has different activity levels and may undergo decomposition to release Si into the overlying water under suitable environmental conditions [20,21]. In this way, it becomes a source of Si in the water, playing a significant role in the biogeochemical Si cycles in aquatic ecosystems. Valid-Si can reflect the primary productivity of aquatic ecosystems, indicate the dynamic succession of algae in eutrophic lakes, and promote the growth of large aquatic plants. The biogeochemical cycles of biogenic elements such as carbon and nitrogen in lake sediments have been extensively studied, but there are few reports on Si in lake environments, especially its chemical species, potential activity, and release characteristics in sediments [7]. This has greatly limited the scientific understanding of the biogeochemical cycles of Si in lake ecosystems, as well as of the coupling relationships between biogenic elements such as carbon, nitrogen, and Si.

This study examined the different silicon forms and BSi distribution characteristics of Valid-Si in the surface sediments of a macrophytic eutrophic lake. By conducting a coordinated analysis of total organic carbon (TOC), total nitrogen (TN), and Chlorophyll-a in the sediments, the impacts of the biogeochemical cycles of Si on the aquatic environment were explored along with its coupling relationships with carbon (C) and nitrogen (N), providing a basis for deepening the understanding of biogeochemical behavioral relationships among different typical biogenic elements (such as Si, C, N) in the lake aquatic ecosystem, as well as the dynamic evolution and environmental protection of macrophytic eutrophic lakes.

## 2. Materials and Methods

### 2.1. Research Area and Sample Collection

Caohai Lake (26°47′–26°52′ N, 104°10′–104°20′ E) is the largest natural freshwater lake and a typical karst lake in Guizhou Province of China, with an average water depth of 1.5 m and a maximum water depth of about 5 m [22]. The climate type in the Caohai Lake region is a subtropical monsoon climate with an average annual temperature of 10.6 °C, as well as an average annual rainfall of 910 mm [23]. Caohai Wetland is a national nature reserve in China, rich in biological resources such as fish, shrimps, and birds. It is an important wintering habitat and a migration station for a variety of rare migratory birds (such as *Grus nigricollis*, *Grus japonensis*, *Ixobrychus sinensis*, *Himantopus himantopus*, and *Ardea purpurea*) [24]. Because of its abundant aquatic plants and macroalgae, Caohai Lake has become one of the most typical macrophytic lakes in China. In recent years, the peripheral areas of Caohai Lake have experienced rapid urbanization; due to the insufficiency of investment in sewage treatment, its nearby villages and towns are now facing a series of serious environmental problems, such as agricultural non-point source pollution and domestic sewage discharge. The ecological environment of Caohai Lake has degraded significantly, and the nutrients in some lake areas have exceeded relevant standards, resulting in eutrophication, deteriorated water quality, and reduced biodiversity. The submerged plants that used to be extremely abundant have disappeared on a large scale, and some areas have gradually shifted from a macrophytic “clear-water state” to an algae-type “muddy-water state” [25]. Many studies have been conducted on carbon, nitrogen, phosphorus, and heavy metal pollution in the water of Caohai Lake, but there has been no public report on Si [22,26,27,28].

**Figure 1:**
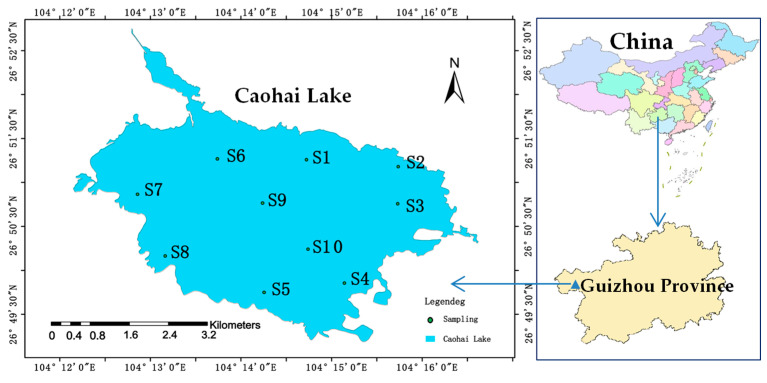
Sediment sampling sites of Caohai Lake.

In January 2022, it was winter and the average value of chemical indexes of Caohai Lake water were generally as follows: the pH was 8.4, the temperature was 8.8 °C, the dissolved oxygen (DO) was 7.9 mg/L, the electrical conductivity (EC) value was 40.0 mS/m, and total phosphorus (TP), total nitrogen (TN) and Chlorophyll-a were 0.031 mg/L, 1.92 mg/L, 14.9 μg/L, respectively [23]. A total of ten representative sites (S1–S10) in Caohai Lake were selected (Figure 1) for the collection of surface sediment samples (around 10 cm) using a Petersen grab sampler. The samples were then refrigerated, quickly transported back to the laboratory, and subjected to treatments such as lyophilization, grinding, and sieving (100-mesh sieve). After treatment, they were cryopreserved at −20 °C for later use.

### 2.2. Extraction and Determination of Si in Sediments

#### 2.2.1. Sequential Extraction of Different Si Forms

**Figure 2:**
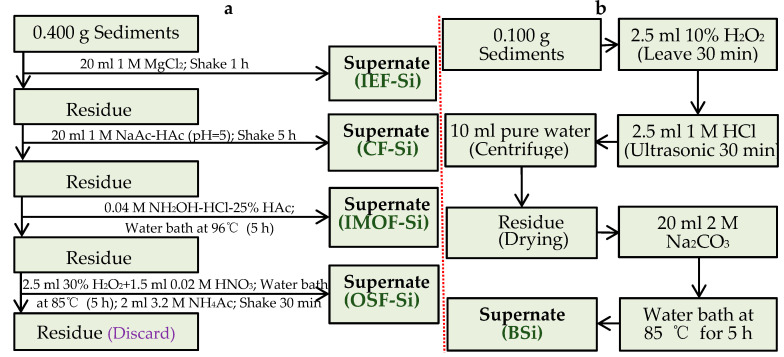
Extraction steps of different silicon (Si) forms (**a**) and biogenic silicon (BSi) (**b**) in sediments.

The different Si forms of Valid-Si in sediments include ion-exchangeable silicon (IEF-Si), carbonate-bonded silicon (CF-Si), iron–manganese-oxide-bonded silicon (IMOF-Si), and organic sulfide-bonded silicon (OSF-Si). Based on the modified Tessier extraction method, the extraction steps were as follows [21,29,30]: A sample of 0.400 g sediment was weighed into a centrifuge tube, 20 mL of 1 M MgCl_2_ was added, it was continuously oscillated for 1 h and then it was centrifuged to obtain IEF-Si supernatant. Following this, 20 mL of a 1 M NaAc-HAc solution (pH = 5) was added to the centrifuged residue and incubated for 5 h of continuous oscillation; the CF-Si supernatant was extracted by centrifugation. Afterward, 20 mL of a 0.04 M NH_2_OH-HCl-25% HAc solution was added to the centrifuged residue and incubated in a water bath at 96 °C for 5 h; then, it was centrifuged to obtain the IMOF-Si supernatant. Finally, 2.5 mL of 30% H_2_O_2_ and 1.5 mL of 0.02 M HNO_3_ were added to the centrifuged residue and, after the reaction subsided, was incubated in a water bath at 85 °C for 5 h. After cooling, 2.0 mL of 3.2 M NH_4_Ac was added and oscillated for 30 min at room temperature; then, it was centrifuged to obtain the OSF-Si supernatant (Figure 2a). We set three parallel groups.

#### 2.2.2. Extraction of BSi

A sample of 0.100 g of the sediment was weighed, and 2.5 mL of a 10% H_2_O_2_ solution was added; the mixture was allowed to stand for 30 min. Then, 2.5 mL of 1 M HCl was added, followed by 30 min of ultrasonic oscillation. Next, 10 mL of pure water was added and the sample was centrifuged before the supernatant was discarded. After the residue was lyophilized, 20 mL of 2 M Na_2_CO_3_ was added, followed by 30 min of ultrasonic oscillation. The mixture was then heated in an 85 °C water bath for 5 h (stirred at intervals of 2 h) and centrifuged to collect the BSi supernatant (Figure 2b) [30]. We set three parallel groups.

It should be noted that glass containers were used as little as possible to prevent the introduction of external Si impurities during these extraction steps, which can lead to errors in Si determination results. The contents of Si in the supernatant of the different Si forms and BSi were determined at a wavelength of 812 nm by silicon–molybdenum blue spectrophotometry [31,32].

### 2.3. Determination of TOC, TN, and Chlorophyll-a in Sediments

After the sediment sample was soaked in 1 M HCl for 24 h and lyophilized, 0.300 g was weighed into a tin vessel, and the contents of TOC and TN were determined using high-temperature combustion with an Elementar element analyzer.

In as little light as possible, a sample of 0.050 g of the sediment was weighed into a centrifuge tube with 5 mL of an acetone–formaldehyde–water mixture (volume ratio 45:45:10) and placed in a 0 °C ice–water mixture for 24 h of ultrasonic extraction. Afterward, it was immediately centrifuged, and the supernatant was used to measure the absorbance at wavelengths of 649 and 665 nm by spectrophotometry to calculate the content of Chlorophyll-a [30,33]. We set three parallel groups.

### 2.4. Data Analysis

Excel 2010, SPSS 2022, and Sigmaplot 10.0 were used in data sorting, statistics, and plotting, respectively. The data were expressed as means and standard deviations of parallel samples. Significant differences and correlation in the data were analyzed using single-factor ANOVA with LSD and Pearson correlation, respectively.

## 3. Results and Discussions

### 3.1. Content Characteristics of Different Si Forms in Caohai Lake Sediments

The different Si forms of Valid-Si in sediments differ greatly in terms of origins and activity and generally have different biogeochemical characteristics. IEF-Si is weakly adsorbed Si with high activity; it can be directly dissolved or decomposed by ion exchange as a result of changes in the sediment–water interface (such as temperature, pH, and disturbance), and released into the overlying water then participates in biogeochemical Si cycles in the water [13,14,21]. CF-Si mainly involves Si bonded with carbonates through co-precipitation and also has high activity, especially when released into the overlying water with the dissolution of carbonates under acidic conditions [34]. IMOF-Si refers to Si bonded with iron–manganese oxides, mainly controlled by the redox conditions at the sediment–water interface. Under oxidizing conditions, low-valent Fe^2+^ and Mn^2+^ are oxidized into iron–manganese oxides, which can continuously adsorb and bond water-soluble Si; in turn, under anoxic reduction conditions, with the reduction of Fe^3+^ into Fe^2+^ and of Mn^4+^ into Mn^2+^ at the sediment–water interface, Si can be released into the overlying water with Fe and Mn [13,21,35,36]. OSF-Si refers to Si bonded with organic sulfides; compared with the other three forms, it is more stable, less active, and less likely to be released in the short term. However, as the organic matter in the sediments is mineralized and decomposed over time, it can be gradually released into the overlying water [37,38,39].

**Figure 3:**
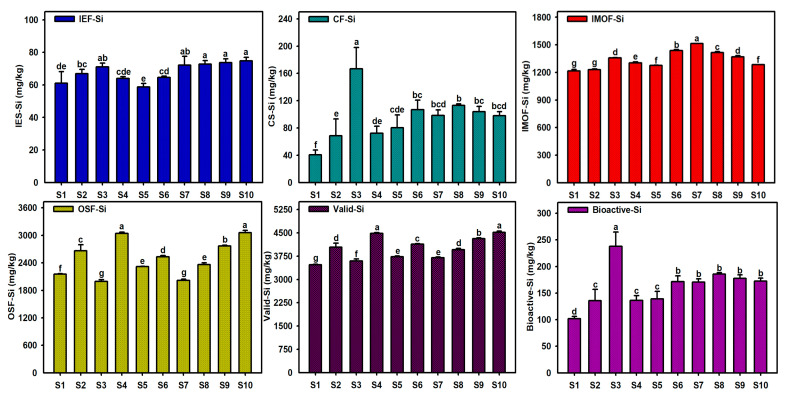
Contents of different Si forms in sediments of Caohai Lake. (Note: The different letter indicated that there was significant difference in the contents of same Si form at different sediment site (*p* < 0.05)).

**Figure 4:**
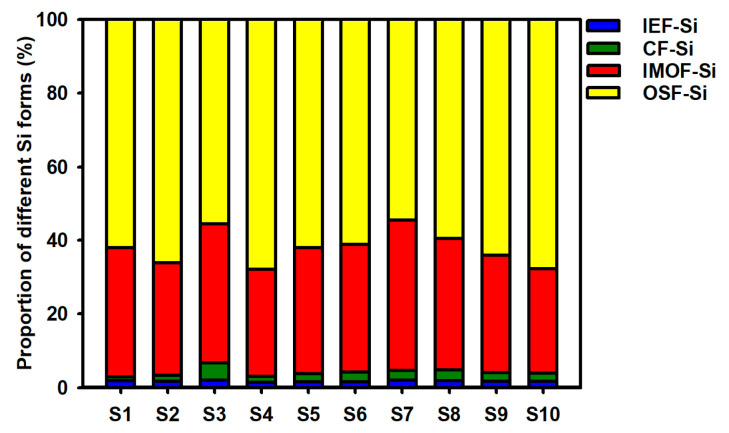
Proportions of different Si forms in sediments of Caohai Lake.

**Figure 5:**
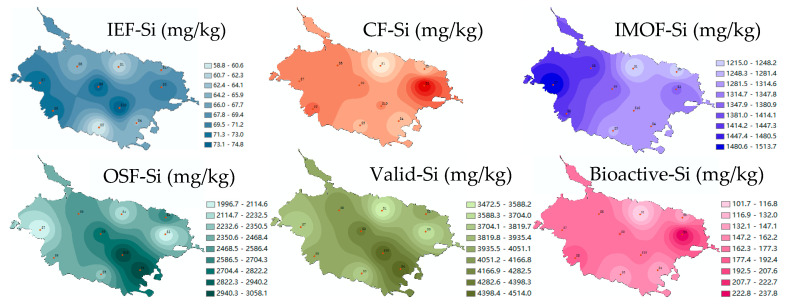
Spatial distribution of different Si forms in sediments of Caohai Lake.

As shown in Figure 3, Figure 4 and Figure 5, the different Si forms in the sediments at different sites in Caohai Lake differed greatly in content. The content of IEF-Si in the sediments was low, ranging between 58.8 and 74.8 mg/kg, with an average of 68.0 mg/kg. Overall, the differences between different lake areas were small, though the content of IEF-Si was relatively higher in the central and western areas (S7–S10). The content of CF-Si in the sediments ranged from 40.6 to 166.7 mg/kg, with an average of 94.9 mg/kg, and it was the highest in the east (S3) and the lowest in the north (S1). The content of IMOF-Si was generally high, ranging from 1215.0 to 1513.7 mg/kg, with an average of 1340.3 mg/kg. The content of IMOF-Si in the west (S7) was significantly higher than that in any other areas, and it was relatively lower in the north (S1) and northeast (S2). Among the four Si forms, OSF-Si had the highest content, ranging from 1996.6 to 3043.1 mg/kg, with an average of 2491.0 mg/kg. The content of OSF-Si near the southwestern and central areas (S4, S9, S10) was significantly higher than that in any other area, and in the east (S3) and west (S7), it was significantly lower. The content of Valid-Si in the sediments at different sites of Caohai Lake ranged from 3472.4 to 4514.3 mg/kg, with an average of 3995.4 mg/kg. Consistent with OSF-Si, the content of Valid-Si was higher near the southwestern and central areas (S4, S10, S9). As IEF-Si and CF-Si have the strongest bioavailability, they are collectively referred to as Bioactive-Si, the content of which was calculated to be 101.7–237.8 mg/kg, with an average of 162.9 mg/kg, and in the east (S3), it was significantly higher than that at any other site, while it was the lowest in the north (S1) and similar with little difference between the central and western areas (S6–S10, *p* > 0.05).

Caohai Lake is located in a typical karst area in southwest China. The strata in the basin are dominated by carbonate rocks, which are supplemented by silicate minerals (such as siliceous rocks and quartz sand) and thin coal seams (containing gypsum sulfur) [40]. The formation of Caohai Lake is inseparable from the weathering of carbonate rocks and silicate minerals. In nature, CO_2_ can form carbonic acid with water, which have a dissolution effect on silicate minerals. A large amount of CO_2_ has been discharged from anthropogenic activities, which also affect the dissolution and weathering of silicate minerals in the basin [41]. These weathered products enter the lake in the form of siliceous particles or dissolved silicates along with surface runoff, serving as an initial source of Si. Overall, the four different Si forms of Valid-Si in surface sediments at different sites in Caohai Lake could be ranked as follows in descending order of content: OSF-Si > IMOF-Si > CF-Si > IEF-Si (Figure 4). The total content of IEF-Si and CF-Si was low, accounting for only 2.9–6.6% (4.1% on average) of Valid-Si. However, both IEF-Si and CF-Si are Bioactive-Si, with great release potential and high bioavailability, and they can become the preferred contributors in the Si biogeochemical cycle. With the intensified decline of submerged plants in Caohai Lake in recent years, the sediment–water interface has become increasingly susceptible to decomposition and acidification, increasing the risks of CF-Si dissolution and release. On average, the content of IOMF-Si and OSF-Si accounted for 33.8%, 62.1%, respectively. The total reached 95.9% of Valid-Si in the surface sediments of Caohai Lake, which indicated that the adsorption and fixation of Si by organic matter and iron–manganese oxides constituted the main mechanism underlying the formation of the different Si forms of Valid-Si in sediments. It also indicated that OSF-Si and IOMF-Si were long-time potential contributors of endogenous Si in Caohai Lake water. In recent years, the eutrophication of Caohai Lake has intensified, presenting a significant trend to an algae-type “muddy-water state” in some areas. Algae have grown too fast, making it easy for a relatively anoxic environment to form at the sediment–water interface. It can promote the reduction and dissolution of iron–manganese oxides and cause the release of IMOF-Si, which may serve as a major contributor to endogenous Si during periods of intensified eutrophication [42,43]. Although it has the lowest activity, OSF-Si is the largest Si pool (54.5–67.9% of Valid-Si) and can continuously release Si with the mineralization and degradation of organic matter in sediments, exerting long-term impacts on the primary productivity of the overlying water and the biogeochemical cycles of Si in the aquatic ecosystem. Comparing this study and our previous work on Dianchi Lake (a typical algae-type eutrophic lake in China) [30], both lakes belong to karst areas, and the initial sources of silicon include natural or anthropogenic silicate minerals weathering products, but the fate of silicon after entering the lake water is different. The two lakes are similar in terms of the content of IEF-Si, CF-Si, and Bioactive-Si in the sediments (bioavailability of Si) but differ significantly in the content of IMOF-Si and OSF-Si, and the content of Valid-Si in Caohai Lake sediments is slightly lower than that in Dianchi Lake sediments. These results demonstrate the significant differences between the two types of lakes in the formation mechanism, potential release, and recycling characteristics of sedimentary Si forms.

### 3.2. Content Characteristics of BSi, TOC, TN, and Chlorophyll-a in Sediments of Caohai Lake

**Figure 6:**
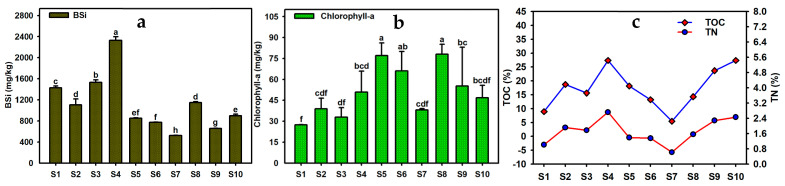
Contents of BSi (**a**), Chlorophyll-a (**b**), and TOC and TN (**c**) in Caohai Lake sediments. (Note: The different letter indicated that there was significant difference in the contents of BSi, Chlorophyll-a at different sediment site, respectively (*p* < 0.05)).

**Figure 7:**
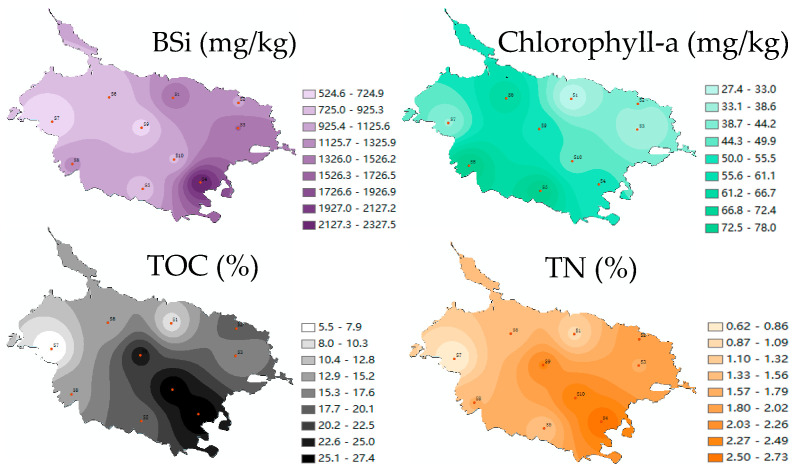
Spatial distribution of BSi, Chlorophyll-a, TOC, and TN in Caohai Lake sediments.

The content of BSi in the surface sediments of Caohai Lake ranged from 524.5 to 2327.9 mg/kg, with an average of 1124.9 mg/kg (Figure 6a). There were significant differences between areas in the content of BSi. The content of BSi was high in the southeast (S4), east (S3), as well as some of the north region (S1) and relatively low in the midwest (S7, S9) and northwest (S6) (Figure 7). BSi comes from the deposition of the shells of siliceous organisms (mainly diatoms), and it is often closely related to the primary productivity of waters, playing an indicative role in the dynamic succession and spatial abundance of lake diatoms [44]. Since the eastern part of Caohai Lake belongs to the upper reaches and is close to Weining County, it is influenced by urban industrial and domestic sewage; its southern part is affected by intensive agricultural activities, and its southeastern part is impacted by the White Horse River, a major inflowing river of Caohai Lake. As a result, the water pollution of Caohai Lake is generally more serious in the southeast and east than in the west and northwest, as embodied in the more obvious eutrophication and the higher algal abundance. Samples for this study were collected in the winter, a season of increased diatom abundance [23]. The post-extinction deposition of diatoms has potentially contributed to the higher content of BSi in the sediments in the southeastern and eastern parts of Caohai Lake. Compared with Dianchi Lake and three non-eutrophic reservoirs in the same region, the content of BSi in Caohai Lake sediments is relatively lower, which is consistent with the fact that Caohai Lake is generally dominated by aquatic plants, and planktonic algae (diatoms) and other siliceous organisms have relatively low abundances and are mainly concentrated in small local areas [30,45].

The content of TOC in the surface sediments of Caohai Lake was high, ranging from 5.5% to 27.4% (17.3% on average), and showed significant differences between sites (Figure 6c). The content of TOC in the sediments exceeded 20% at S4, S10, and S9 (i.e., near the southwestern and central areas) but was only 5.5% and 8.9% at S7 (west) and S1 (north), respectively. Similarly, the content of TN in the surface sediments of Caohai Lake was also high, ranging from 0.6% to 2.7% (1.7% on average), and it showed significant differences between sites; it exceeded 2% at S4, S9, and S10 but was only 0.6% and 1.0% at S7 and S1, respectively. Over the years, aquatic organisms such as submerged plants, planktonic animals/plants, and algae have grown well in Caohai Lake, endowing it with high biodiversity. The extinction and deposition of aquatic organisms have accumulated abundant organic matter, causing the contents of TOC and TN in Caohai Lake to be significantly higher than that in many famous lakes such as Taihu Lake, Poyang Lake, and Ulansuhai Nur Lake, as well as Hongfeng Lake, Baihua Lake, and Dianchi Lake in the same basin [30,46]. Spatially (Figure 7), TOC and TN in the sediments were relatively consistent, declining from the southeast, south, and east to the north and west, which was related to the heavy pollution in the eastern and southern parts of the lake. Besides the contribution of endogenous organic matter in Caohai Lake water, there was also a substantial input of exogenous organic matter [46]. The content of Chlorophyll-a in the sediments of Caohai Lake ranged from 27.4 to 78.0 mg/kg (51.2 mg/kg on average), and the content of Chlorophyll-a at S8, S5, and S6 was higher than that at any other site (Figure 6b). Spatially, the content of Chlorophyll-a was relatively high in the south, southwest, middle, and northwest but relatively low in the east, northeast, and west, which comprehensively reflected the spatial distribution, extinction, and deposition characteristics of aquatic plants and algae in Caohai Lake [47].

### 3.3. Indicative Significance of C/N, C/Si, N/Si, and BSi/Bioactive-Si in Caohai Lake Sediments

**Figure 8:**
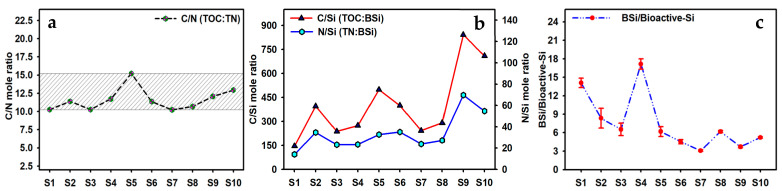
Mole ratio of C/N (**a**), C/Si (**b**), N/Si (**b**), and BSi/Bioactive-Si (**c**) in Caohai Lake sediments.

The C/N mole ratio of TOC to TN in sediments can effectively indicate the sources of organic matter. Since terrestrial plants are rich in cellulose and lignin, their C/N mole ratios are often higher than 20; in contrast, lake endophytes are rich in proteins, so their C/N mole ratios are relatively low, often below 10 [48,49]. The C/N mole ratio in the surface sediments of Caohai Lake ranged between 10.2 and 15.2 (11.6 on average) (Figure 8a). It changed little (10.2–12.9) across different sites, except for S5 (15.2), which indicated that the sources of organic matter in the sediments of Caohai Lake included both the residues of endophytes (the main contributors) and the input of terrigenous organic matter. S5 is close to agricultural areas, so rainfall scouring and leaching might have significantly increased the input of terrigenous soil organic matter [46].

The C/Si mole ratio of TOC to BSi and the N/Si mole ratio of TN to BSi can comprehensively reflect the preservation rates of organic matter and BSi in the sediments [50]. In the surface sediments of Caohai Lake, the C/Si mole ratio ranged between 146.0 and 842.0 (403.7 on average), and the N/Si mole ratio ranged from 14.2 to 69.7 (33.9 on average); both far exceeded the famous Redfield ratio (C:N:Si = 106:16:16; C/Si = 6.625; N/Si = 1) [6,33,50] (Figure 8b). This is related to the fact that the contents of TOC and TN were much higher than that of BSi in the sediments of Caohai Lake, and it suggests that the dissolution and release rate of BSi may be much higher than the degradation rate of organic matter in the sediments [50,51,52]. In particular, in the eastern and southern areas (sites S2~S5), because of the very good correlation between BSi, TOC, and TN (*p* < 0.01, Table 1), that is, they belong to the same source (i.e., mainly the residues of endophytes, such as diatoms, based on the C/N mole ratio) with high probability, the original mole ratios of C/Si and N/Si were probably close to the Redfield ratio or the C/Si and N/Si mole ratio of diatoms at the beginning, and gradually, BSi was likely to dissolve at a higher rate and more rapidly participate in the biogeochemical cycles of Si in the overlying water than the degradation rate of organic matter, resulting in a high mole ratio of C/Si and N/Si in the lake sediments [52,53]. Caohai Lake water is weakly alkaline, playing a significant role in promoting the release and dissolution of BSi in the surface sediments [40]. The content ratio of BSi/Bioactive-Si can reflect the conversion from Bioactive-Si to BSi; a high ratio implies the continuous dissolution of Bioactive-Si and its utilization by siliceous organisms for increased storage and accumulation of BSi [54]. In the surface sediments of Caohai Lake, the content ratio of BSi/Bioactive-Si ranged from 3.1 to 17.1 (7.5 on average), and it was the highest and lowest in the southeast (S4) and the west (S7), respectively (Figure 8c). Overall, it was higher in the northern, eastern, and southern areas (S1–S5) than in the central and western areas (S6–S10), which indicated that, in the sediments in the north, east, and south of Caohai Lake, BSi was continuously accumulated while Bioactive-Si was released and utilized [20].

### 3.4. Correlations of Different Si Forms with BSi, TOC, TN, and Chlorophyll-a in Caohai Lake Sediments

Based on the spatial distribution of aquatic organisms and the pollution characteristics of its water environment, Caohai Lake was divided into two zones: the algae-type eutrophic zone in the eastern and southern areas (S2/S3/S4/S5) and the macrophytic clear-water zone in the western, middle, and northern areas (S1/S6/S7/S8/S9/S10). The correlations of the different Si forms with BSi, TOC, TN, and Chlorophyll-a in the sediments were analyzed. This zoning method could relatively scientifically explain the internal relationships between the biogeochemical behaviors of different substances (such as Si, C, N, Chlorophyll-a) in the sediments of Caohai Lake.

As shown in Table 1, IEF-Si, CF-Si, and Bioactive-Si were significantly positively correlated with each other in each zone, suggesting that there might have been a common mechanism underlying the sources of CF-Si and IEF-Si and that they jointly dominated Bioactive-Si. There was a highly significant positive correlation between CF-Si and IMOF-Si in each zone (*p* < 0.01), which also indicated the presence of a good coupling relationship between them in terms of sources. The significant negative correlation between CF-Si and OSF-Si in the algae-type eutrophic zone demonstrated the trade-off coupling between the two Si forms. All these suggested that the different Si forms in Caohai Lake sediments might have been simultaneously influenced by the direct transport and deposition of terrigenous (homologous) Si and the competitive adsorption and fixation of dissolved Si in different water phases. This created a sharp contrast with the poor coupling relationship between the different Si forms of Valid-Si previously seen in the Dianchi Lake sediments, indicating the presence of great differences in the biogeochemical behavior and cycling mechanisms of Si between two different types of lakes. In particular, given that OSF-Si occupied the highest content in the Caohai Lake sediments and had highly significant positive correlations with Valid-Si, TOC, and TN in each zone (*p* < 0.01), it plays a significant role in determining the content change of Valid-Si in Caohai Lake sediments, also indicating that Si has tight coupling with carbon and nitrogen.

There was a highly significant positive correlation between TOC and TN in Caohai Lake sediments of each zone (*p* < 0.01) (Table 1), and the δ^13^C and δ^15^N values of the sediments were −24.48‰~−20.53‰ and 1.70‰~4.21‰ (consistent with aquatic plants and terrestrial soil organic matter), respectively [55], indicating that TOC and TN had high homology. TN was dominated by organic nitrogen, that is, due to the relatively stable content of carbon and nitrogen in organisms, their residues decompose to form organic matter in sediments [56,57]. In the sediments of the algae-type eutrophic zone, based on the eastern and southern areas, BSi had highly significant positive correlations with TOC and TN (*p* < 0.01), indicating that there was a significant coupling relationship between the preservation of BSi and the accumulation of organic matter, that is, the deposition of the residues of siliceous organisms made a substantial contribution to organic matter, which testified to the higher abundance of siliceous organisms (such as diatoms) in this zone and the ability of BSi to reflect the primary productivity of diatoms [17,33]. However, in the macrophytic clear-water zone, based on the western, middle, and northern areas, BSi had no correlation with TOC or TN, which reflected the small contribution of the deposition of the residues of siliceous organisms to organic matter in this zone. Overall, these results are consistent with the characteristics of the eastern and southern parts of Caohai Lake (serious eutrophication, massively proliferative algae, and increased diatom abundance in the winter), as well as the characteristics of the western, middle, and northern parts of Caohai Lake (milder eutrophication, a higher abundance of large submerged plants, and a lower proportion of siliceous organisms such as diatoms).

Finally, regardless of the type of lake, with the continuous control of exogenous pollution, the regulation effect of substances inside the lake on the water ecosystem has been further enhanced. Obviously, as the significant source inside the lake, the release of various substances in sediments profoundly affect the substance balance of overlying water and then affect the stability of the lake water ecosystem. Therefore, it is necessary to further explore and pay attention to the release mechanism of valid silicon in sediments, as well as the coupling relationship, activity difference, and synchronous release mechanism between silicon and other biogenic elements such as carbon (C), nitrogen (N), and phosphorus (P) in sediments, because the release and recycling of these biogenic elements from sediments can easily break the stoichiometric balance of different biogenic elements in overlying water, which can regulate the diversity and abundance of different plankton species, thereby affecting the balance of aquatic ecosystems and creating potential safety risks [58,59,60]. Moreover, the current global warming and the frequent occurrence of regional extreme weather events undoubtedly have many unpredictable impacts on lake water ecosystems and various substance cycles of lakes; in particular, they may enhance the migration and transformation of lake ecosystem substances. For example, high-temperature weather can accelerate the chemical reactivity of the substances in the lake and affect the release activity at the sediment–water interface [61], and strong storms can significantly disturb the surface sediment and improve the internal substances’ re-suspension [62]. In general, under changing external environmental conditions, the biogeochemical characteristics and release behavior of silicon and its coupling relationship with other biogenic elements (e.g., C, N, P) in lake sediments deserve long-term attention.

## 4. Conclusions

We systematically report, for the first time, the content characteristics of different Si forms, BSi, TOC, and TN in surface sediments from Caohai Lake, a typical macrophytic lake in China, as well as their coupling relationships. The different Si forms of Valid-Si in the surface sediments of Caohai Lake could be ranked as follows in descending order of content: OSF-Si > IMOF-Si > CF-Si > IEF-Si, with OSF-Si (62.1%) and IOMF-Si (33.8%) jointly accounting for 95.9%. This indicated that the fixation of Si by organic matter and iron–manganese oxides constituted the main mechanism underlying the formation of the different Valid-Si chemical species in the sediments. Meanwhile, the release of Si from the sediments and its recycling might have been driven mainly by the mineralized degradation of organic matter and the anoxic reduction conditions at the sediment–water interface. The content of BSi in the sediments of Caohai Lake was relatively high in the southeastern, eastern, and northern areas, which could be explained by the intensification of eutrophication and the increased abundance of diatomaceous siliceous organisms in these areas in recent years. The contents of TOC and TN in the sediments of Caohai Lake were generally high, with a C/N mole ratio of 10.2–15.2, which indicated that the sources of the organic matter in the sediments included both the residues of endophytes (the primary contributors) and the input of terrigenous organic matter. In addition, TOC and TN in the sediments of Caohai Lake both had highly significant correlations with OSF-Si and Valid-Si, which demonstrated that Valid-Si in the sediments had excellent coupling relationships with C and N. Because of the good correlation between BSi, TOC, and TN (*p* < 0.01), as well as the high C/Si and N/Si mole ratios of TOC and TN to BSi, respectively, they probably belong to the same source, i.e., mainly the residues of endophytes, such as diatoms, and BSi may have faster dissolution and release characteristics than organic matter from sediments in lake areas with a higher abundance of siliceous organisms. This study provides basic information for us to understand the biogeochemical behavior of Si and its correlation with other typical biogenic elements such as carbon and nitrogen in lake aquatic ecosystems.

## Figures and Tables

**Table 1:** Correlations of different Si forms with BSi, TOC, TN, and Chlorophyll-a in Caohai Lake sediments.

Name	IEF-Si	CF-Si	IMOF-Si	OSF-Si	Valid-Si	Bioactive-Si	BSi	Chlorophyll-a	TN	TOC
IEF-Si	1	0.527 *	0.269	0.398	0.529 *	0.671 **	−0.448	0.242	0.455	0.469 *
CF-Si	0 .637 *	1	0.696 **	0.341	0.618 **	0.984 **	−0.637 **	0.708 **	0.336	0.327
IMOF-Si	0 .402	0 .755 **	1	−0.324	0.011	0.664 **	−0.733 **	0.363	−0.366	−0.362
OSF-Si	− 0 .280	− 0 .720 *	− 0 .471	1	0.942 **	0.382	−0.160	0.226	0.956 **	0.973 **
Valid-Si	− 0 .166	− 0 .596	− 0 .316	0 .983 **	1	0.651 **	−0.419	0.383	0.887 **	0.904 **
Bioactive-Si	0 .696 *	0 .997 **	0 .745 **	− 0 .694 *	− 0 .567	1	−0.651 **	0.669 **	0.389	0.384
BSi	0 .240	0 .040	0 .434	0 .588	0 .713 ^*^	0 .062	1	−0.115	−0.073	−0.120
Chlorophyll-a	− 0 .786 **	− 0 .338	− 0 .271	0 .122	0 .009	− 0 .394	− 0 .301	1	0.270	0.218
TN	0 .163	− 0 .245	0 .079	0 .804 **	0 .889 **	− 0 .212	0 .924 **	− 0 .302	1	0 .994 **
TOC	− 0 .301	− 0 .534	− 0 .124	0 .932 **	0 .956 **	− 0 .528	0 .766 **	0 .138	0 .876 **	1

Note: The dark red data represent the algae-type eutrophic zone (S2/S3/S4/S5); The dark blue data represent the macrophytic clear-water zone (S1/S6/S7/S8/S9/S10). “*” and “**” represent *p* < 0.05 and *p* < 0.01, respectively, for Pearson correlation analysis.

## Data Availability

The original contributions presented in this study are included in the article; further inquiries can be directed to the corresponding authors.
